# Supplementary material for: Palliative Radiotherapy in Metastatic Breast Cancer Patients on CDK4/6 Inhibitors: Safety Analysis
Source: Cancers (Basel). 2025 Jan 27;17(3):424. doi: 10.3390/cancers17030424 (PMC11816231; doi:10.3390/cancers17030424)
Supplement: Supplementary file 1 [file cancers-17-00424-s001.zip › cancers-3397925-supplementary.pdf]

cancers

MDPI

Supplementary Materials

Palliative Radiotherapy in Metastatic Breast Cancer Patients on CDK4/6 Inhibitors: Safety Analysis

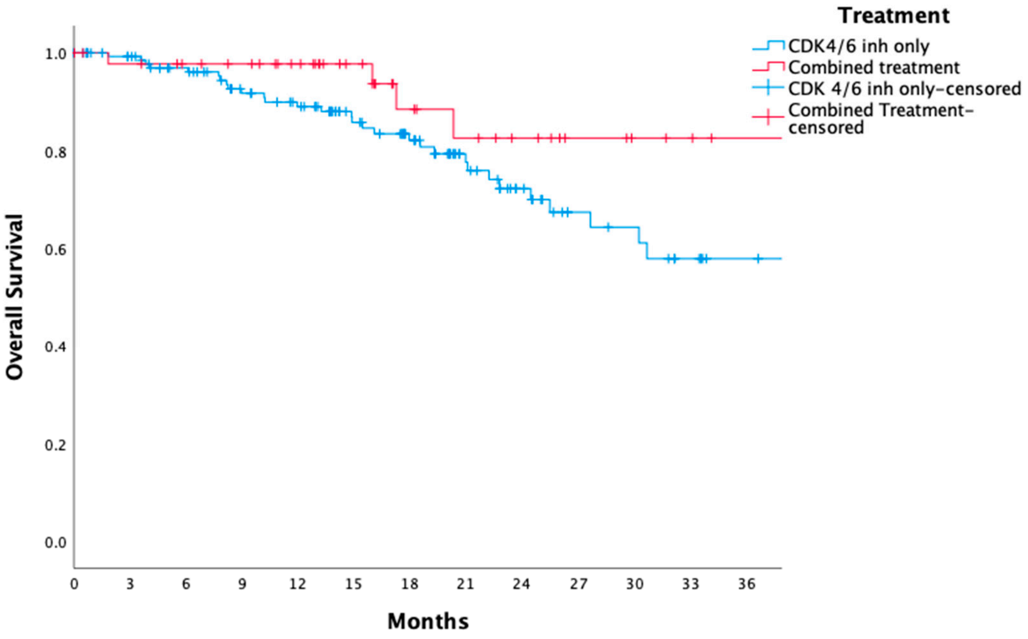

Figure S1. : Kaplan-Meier Survival Analysis of Overall Survival in Patients Treated With CDK4/6 Inhibitors With or Without Concomitant Palliative Radiotherapy. Supplementary Figure S1 presents the Kaplan-Meier survival curve for overall survival (OS) in patients treated with CDK4/6 inhibitors alone versus those receiving CDK4/6 inhibitors in combination with concomitant palliative radiotherapy. Statistical analysis was conducted using the log-rank test, with no significant difference observed between the two groups (41.9 months vs. 39.1 months, p=0.090).

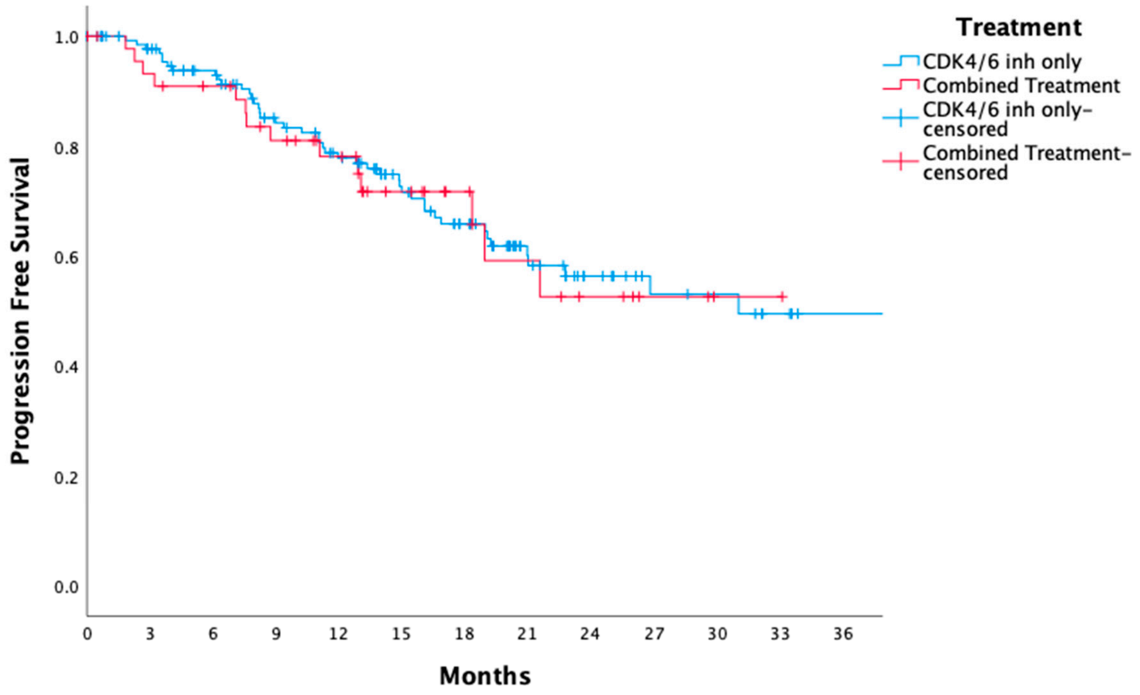

Figure S2. Kaplan-Meier Survival Analysis of Progression-Free Survival in Patients Treated With CDK4/6 Inhibitors With or Without Concomitant Palliative Radiotherapy. Supplementary Figure S2 presents the Kaplan-Meier survival curve for progression-free survival (PFS) in patients receiving CDK4/6 inhibitors alone versus those treated with CDK4/6 inhibitors combined with concomitant palliative radiotherapy. Statistical analysis was performed using the log-rank test, with no significant difference observed between the two groups (not estimable [NE] vs. 31.0 months, p=0.831).

Table S1. Univariate and Multivariate Analysis Results on Grade ≥3 Hematologic Toxicity.

	Univariate Analysis			Multivariate Analysis		
	**OR**	**95% CI**	***p***	**OR**	**95% CI**	***p***
AI (AI vs Fulvestrant)	1.55	0.82-2.92	0.174			
Age (<60 vs ≥60)	0.61	0.34-1.09	0.098			
Ribociclib (Ribociclib vs palbociclib)	0.35	0.19-0.64	<0.001	0.37	0.20-0.70	0.002
First line vs other	0.44	0.22-0.89	0.022	0.52	0.25-1.09	0.082

OR: Odds Ratio; CI: Confidence Interval; AI: Aromatase Inhibitor.

Supplementary Table S1 Univariate and multivariate analysis results on grade ≥3 hematologic toxicity. The table presents the odds ratios (OR), 95% confidence intervals (CI), and p-values (*p*) for various factors influencing the risk of grade ≥3 hematologic toxicity, including the type of endocrine therapy (AI vs. Fulvestrant), age, CDK4/6 inhibitor used (Ribociclib vs. Palbociclib), and line of therapy (first-line vs. others). The results are shown for both univariate and multivariate analyses to identify independent predictors of hematologic toxicity.

Table S2. Comparison of Patient Characteristics Between the Combined Treatment Group (CDK4/6 Inhibitors with Radiotherapy) and the CDK4/6 Inhibitor-Only Group.

		**Whole (*n*=188)**	**Combined (*n*=47)**	**CDKonly (*n*=141)**	***p***
Age, Median (IQR))		57.5 (46-68)	57.0 (44-64)	58 (47-69)	0.333
Gender (Female)		185 (98%)	45 (96%)	140 (99%)	0.093
Menopause	Pre-	38 (20%)	10 (21%)	28 (20%)	

	Post-	147 (78%)	35 (78%)	112 (79%)	0.748
ECOG	0	6 (3%)	1 (2%)	5 (3%)	0.254
	1	163 (86%)	38 (81%)	125 (89%)	
	2	14 (8%)	6 (13%)	8 (6%)	
	3	5 (3%)	2 (4%)	3 (2%)	
HER2	Score 0	143 (79%)	33 (70%)	110 (81%)	0.157
	Score 1	28 (15%)	10 (21%)	18 (13%)	
	Score 2, FISH: Negative	11 (6%)	4 (9%)	7 (6%)	
Metastatic Area	Bone	124 (66%)	32 (68%)	92 (65%)	0.835
	Liver	44 (23%)	10 (21%)	34 (24%)	0.510
	Lung	22 (12%)	7 (15%)	15 (11%)	0.412
	Lymph Node	42 (22%)	13 (28%)	29 (21%)	0.446
	Skin	12 (7%)	2 (4%)	10 (7%)	0.123
Extensive bone metastases		87 (46%)	19 (40%)	68 (43%)	0.237
Vertebral bone metastases		89 (47%)	22 (47%)	67 (48%)	0.736
Received before chemotherapy		72 (37%)	16 (34%)	56 (40%)	0.364
Chemotherapy within one year before CDK4/6		23 (12%)	6 (13%)	17 (12%)	0.833
De Novo Metastatic		119 (63%)	21 (46%)	98 (70%)	0.003
Line	First	143 (76%)	27 (57%)	116 (82%)	0.006
	Second	41 (22%)	17 (36%)	24 (17%)	
	Third and after	4 (2%)	3 (6%)	1 (1%)	
CDK4/6 inhibitors	Palbociclib	71 (38%)	13 (28%)	58 (41%)	0.099
	Ribociclib	117 (62%)	34 (72%)	83 (59%)	
Co-administered Therapy	AI	113 (60%)	25 (54%)	88 (62%)	0.450
	AI + LHRH	18 (10%)	3 (7%)	15 (11%)	
	Fulvestrant	52 (28%)	17 (37%)	35 (25%)	
	Fulvestrant + LHRH	4 (2%)	2 (2%)	2 (2%)	
CDK4/6 inhibitors	Interruption	104 (55%)	25 (56%)	79 (53%)	0.735
	Dose reduction	45 (24%)	13 (28%)	32 (23%)	0.490
	Withdraw	3 (2%)	1 (2%)	2 (1%)	1.000
Progression		62 (33%)	15 (32%)	47 (33%)	0.829
Death		35 (19%)	5 (11%)	30 (21%)	0.053

This table compares the baseline characteristics of patients receiving combined treatment (CDK4/6 inhibitors with radiotherapy) versus those receiving CDK4/6 inhibitors alone. Parameters include demographic data, tumor features, metastatic sites, and treatment details. Data are expressed as frequencies and percentages unless otherwise indicated. CDK4/6: Cyclin-Dependent Kinase 4 and 6, AI: Aromatase Inhibitor, LHRH: Luteinizing Hormone-Releasing Hormone, ECOG PS: Eastern Cooperative Oncology Group Performance Status, PFS: Progression-Free Survival, OS: Overall Survival, SD: Standard Deviation.
